# Risk factors for carbapenem-resistant *Acinetobacter baumannii* (CRAB) bloodstream infections and related mortality in critically ill patients with CRAB colonization

**DOI:** 10.1093/jacamr/dlad096

**Published:** 2023-08-10

**Authors:** Francesco Cogliati Dezza, Sara Covino, Flavia Petrucci, Federica Sacco, Agnese Viscido, Francesca Gavaruzzi, Giancarlo Ceccarelli, Gianmarco Raponi, Cristian Borrazzo, Francesco Alessandri, Claudio Maria Mastroianni, Mario Venditti, Alessandra Oliva

**Affiliations:** Department of Public Health and Infectious Diseases, Sapienza University of Rome, Rome, Italy; Department of Public Health and Infectious Diseases, Sapienza University of Rome, Rome, Italy; Department of Public Health and Infectious Diseases, Sapienza University of Rome, Rome, Italy; Microbiology and Virology Laboratory, Department of Molecular Medicine, Sapienza University of Rome, Rome, Italy; Microbiology and Virology Laboratory, Department of Molecular Medicine, Sapienza University of Rome, Rome, Italy; Department of Public Health and Infectious Diseases, Sapienza University of Rome, Rome, Italy; Department of Public Health and Infectious Diseases, Sapienza University of Rome, Rome, Italy; Microbiology and Virology Laboratory, Department of Molecular Medicine, Sapienza University of Rome, Rome, Italy; Department of Medico-Surgical Sciences and Biotechnologies, Sapienza University of Rome, Rome, Italy; Department of General and Specialistic Surgery, Sapienza University of Rome, Rome, Italy; Department of Public Health and Infectious Diseases, Sapienza University of Rome, Rome, Italy; Department of Public Health and Infectious Diseases, Sapienza University of Rome, Rome, Italy; Department of Public Health and Infectious Diseases, Sapienza University of Rome, Rome, Italy

## Abstract

**Background:**

Among MDR bacteria, carbapenem-resistant *Acinetobacter baumannii* (CRAB) is a major concern due to the limited therapeutic options. During the COVID-19 pandemic, a worrying increase in the spread of CRAB infections was reported.

**Objectives:**

The study assessed the risk factors for CRAB bloodstream infection (BSI) in patients admitted to the ICU with CRAB colonization, and the related mortality risk factors.

**Methods:**

We conducted a single-centre, observational, prospective study; all consecutive patients with CRAB colonization admitted to the ICU of a tertiary hospital in Rome from January 2021 to September 2022 were included in the study. Univariate and multivariate analyses were performed to investigate BSI and mortality risk factors.

**Results:**

Overall, 129 patients were included in the study; 57 (44%) out of these developed BSI. In our study population, at the multivariable analysis the Charlson comorbidity index (CCI) (*P* = 0.026), COVID-19 (*P* < 0.001), multisite colonization (*P* = 0.016) and the need for mechanical ventilation (*P* = 0.024) were risk factors independently associated with BSI development. Furthermore, age (*P* = 0.026), CCI (*P* < 0.001), septic shock (*P* = 0.001) and Pitt score (*P* < 0.001) were independently associated with mortality in the BSI patients. Instead, early appropriate therapy (*P* = 0.002) and clinical improvement within 72 h (*P* = 0.011) were shown to be protective factors.

**Conclusions:**

In critically ill patients colonized by CRAB, higher CCI, multisite colonization and the need for mechanical ventilation were identified as risk factors for BSI onset. These predictors could be useful to identify patients at highest risk of BSI.

## Introduction

The spread of antimicrobial resistance (AMR) is a global emergency that threatens public health worldwide. Also referred to as the ‘hidden pandemic’, nowadays the AMR impact involves not only global health but is playing an increasingly important role in economic and social fields.^1^ In 2018, the WHO published a ranking of MDR bacteria with ‘critical priority’, on which carbapenem-resistant *Acinetobacter baumannii* (CRAB) is in the first place.^2^

CRAB infection has a mortality rate of up to 70%–80% with an increase in length of hospitalization and healthcare costs, especially in patients admitted to ICUs.^3–6^ Whether CRAB infection is an associated or attributable mortality risk factor in critically ill patients has still to be defined; however, when attributable mortality data are reported, the mortality rate in CRAB infections remains very high.^1,7^

Moreover, during the COVID-19 pandemic, there were several nosocomial outbreaks caused by CRAB in ICUs with a significant impact on in-hospital mortality.^8–13^ Among carbapenem-resistant Gram-negative bacilli bloodstream infections (BSIs), CRAB BSIs are the most serious infections with the highest mortality rates.^7^

Differently from carbapenem-resistant Enterobacterales (CRE), where there are well-established clinical risk scores for development of BSI in CRE-colonized patients, for CRAB, little is known about the relationship between colonization and BSI development.^5,14–21^

Appropriate early treatment is one of the most significant factors that may reduce the mortality rate of BSI, as reported for KPC-producing *Klebsiella pneumoniae* BSI.^22^ Therefore, it is plausible that knowing the specific risk factors for BSI development in CRAB-colonized critically ill patients might be crucial to prompt early appropriate therapy aimed to reduce associated mortality.

Based on these considerations, the primary objective of this study was to evaluate risk factors for CRAB BSI in ICU CRAB-colonized patients.

## Materials and methods

### Study design

We conducted a prospective, observational single-centre study in adult patients admitted to the ICUs of a tertiary academic hospital in Rome. From January 2021 to September 2022, all consecutive patients hospitalized in the ICUs with CRAB colonization were included in the study. Inclusion criteria were: (i) age ≥18 years; (ii) admission to ICU; and (iii) colonization by CRAB at any anatomical site. We excluded patients with prior CRAB colonization more than 30 days before the ICU admission, with CRAB BSI without prior colonization, and with expected survival less than 48 h from documented colonization.

**Figure 1.**
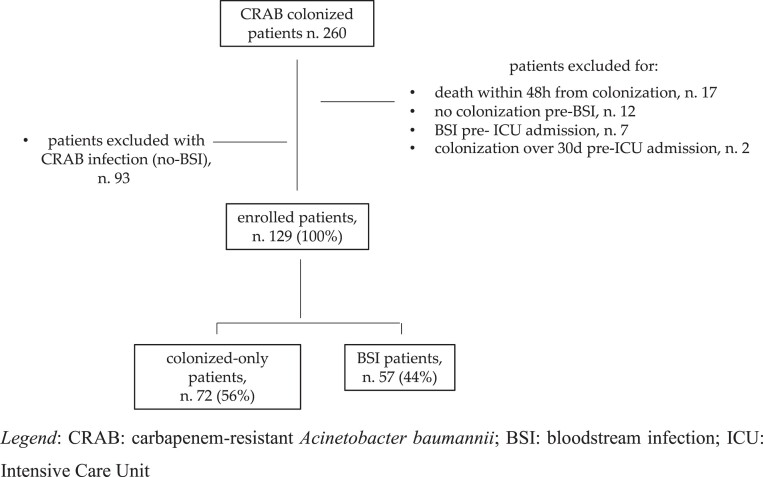
Flow chart of study population.

The study population was further divided into two groups: (i) BSI patients: patients who developed CRAB BSI throughout hospitalization with the same resistance profile of the colonizing pathogen; and (ii) colonized-only patients: patients with CRAB colonization who did not develop BSI or other CRAB infection during the entire hospitalization. Patients with CRAB infections other than BSI were excluded from the study population (Figure 1). The prospective nature of the study was based on the consecutive enrolment of patients. However, all complete data were retrospectively extracted afterwards.

Medical charts were reviewed by trained doctors and the following information was anonymously recorded in an electronic database: demographics, comorbidities, hospitalization/antibiotic therapy/immunosuppression/surgery in the previous 90 days, previous SARS-CoV-2 infection, cause of hospitalization and ICU admission, laboratory and clinical data on the day of colonization and of BSI onset, site of colonization, timing of colonization and BSI onset, time from colonization to BSI onset, microbiological data and antibiotic regimens in the BSI group, duration of ICU and hospital stay, clinical cure, CRAB BSI recurrence, development of new CRAB infection, development of secondary infection, in-hospital mortality and 7 day, 14 day and 28 day mortality from colonization and BSI onset.

### Endpoints

The primary outcomes were CRAB BSI onset and the risk factors associated with BSI development. The secondary outcomes were: (i) mortality from BSI onset at 7 days, 14 days and 28 days and its related predictors in the BSI group; and (ii) the impact of different treatment regimens on 28 day mortality.

### ICU settings

The ICUs at the tertiary hospital in Rome, Policlinico Umberto I, are one general, one dedicated to the emergency department (ER-ICU), one dedicated to neurosurgery (NS-ICU), one dedicated to cardiothoracic surgery (CTS-ICU), one dedicated to transplantation and one dedicated to COVID-19. The beds are distributed as follows: 18 in the general ICU, 6 in the ER-ICU, 9 in the NS-ICU, 6 in the CTS-ICU and 6 in the transplantation ICU, while the number of COVID-19 ICU beds varied according to epidemiological needs during the study period, ranging from 15 to 54.

### Microbiological analyses

The identification of CRAB strains was based accordingly on local laboratory techniques. Blood culture bottles were incubated in the automatic BacT/ALERT Virtuo system (bioMérieux, Inc., Marcy l’Étoile, France). Isolated colonies from blood cultures or other positive cultures were identified using a MALDI-TOF MS system (Bruker Daltonik GmbH, Bremen, Germany). Antimicrobial susceptibility was tested using the MicroScan WalkAway system (Beckman Coulter, Inc., Brea, CA, USA). The determination of cefiderocol susceptibility, when available for the clinicians, was performed with the disc diffusion method. A zone diameter of ≥17 mm for the cefiderocol 30 μg disc corresponded to MIC values below the pharmacokinetic/pharmacodynamic breakpoint of S ≤ 2 mg/L. The MICs of antibiotics were assessed by following EUCAST criteria.^23^

### Definitions

Infections were defined according to the standard definitions of ECDC.^24^ CRAB BSI was defined when CRAB was isolated from blood cultures in the presence of clinical signs of infection and BSI onset was considered the date of the index blood culture collection. Primary BSI was defined as a BSI occurring in patients without a recognized source of infection.

MDR, XDR and pandrug-resistant (PDR) bacteria were defined according to the classification of Magiorakos *et al*.^25^

The burden of comorbidities was estimated by means of the Charlson comorbidity index (CCI), while patients’ severity at ICU admission was defined by the SAPS II score.^26,27^ The severity of BSI was defined according to the Pitt bacteraemia score at BSI onset.^28^ Septic shock was defined according to the international consensus.^29^

Immunosuppression was defined as either steroid therapy with prednisone (or its equivalent) at a dose of >0.5 mg/kg/day for at least 1 month or the receipt of chemotherapy, TNF-α inhibitors, cyclophosphamide, azathioprine, methotrexate or mycophenolate mofetil in the previous 90 days.

The source control was achieved when all those physical measures used to control the focus of BSI and to restore the optimal function of the affected area were performed.

According to the hospital’s guidelines for ICUs, rectal/stool swabs, respiratory and urine cultures were performed at ICU admission and routinely re-evaluated once a week for MDR organism (MDRO) strains; in the other hospital settings, regular MDRO screening has not been recommended unless suggested for clinical or epidemiological reasons. Screening for Gram-negative MDROs by throat and skin swabs is not performed in our hospital. Colonization was defined as a positive culture of a specimen from any anatomical site in the absence of clinical signs of infection. Multisite colonization was defined as a positive culture from more than one specimen from different anatomical sites in the same patient during the ICU hospitalization before BSI onset.

The differences between CRAB colonization and infection, in sites other than rectal swab isolation, were evaluated case by case according to patients’ clinical conditions, presence of signs/symptoms or laboratory parameters suggestive of infections and the eventual targeted treatment chosen by the dedicated Infectious Disease consultants. In case of doubt, a shared discussion was made case by case.

### Appropriateness of therapy

Early appropriate therapy was defined as the use of at least one *in vitro* active drug within the first 24 h from BSI onset, definitive antibiotic therapy was defined as the specific antimicrobial treatment administered after the availability of susceptibility tests, and the time to definitive therapy was the number of days from BSI onset to the definitive therapy. Definitive antibiotic therapy was considered appropriate if the CRAB strain isolated was susceptible to the chosen drug regimen.

### Outcomes

BSI onset was considered as a CRAB BSI occurring during hospitalization.

Clinical improvement at 48–72 h after initiation of antibiotic therapy was defined as at least one of the following: discontinuation of treatment with inotropic drugs if the patient was in septic shock, or disappearance of fever for at least 48 h, or reduction of serum procalcitonin values by at least 80% compared with initial value/achievement of a procalcitonin value of <0.5 ng/mL, or reduction of at least 75% of the maximum C-reactive protein (CRP) value achieved.^30^

Clinical cure was the resolution of symptoms after discontinuation of antibiotic therapy, whereas microbiological positive outcome was defined as negative follow-up blood cultures at 72 h, 7 days or 14 days after the start of antibiotic treatment.

CRAB BSI recurrence was defined as the onset of a second microbiologically documented CRAB BSI in the 30 days after the end of treatment in a patient who had previously achieved a clinical cure and microbiological positive outcome.

New CRAB infection was considered as isolation of CRAB causing infections other than BSI in the 30 days after the end of treatment and achieving a clinical cure.

Secondary infection was defined as an infection caused by an MDRO other than CRAB in the 30 days after the start of treatment for BSI.

All-cause mortality at 7, 14 and 28 days after documented colonization and BSI onset was recorded.

### Statistical analyses

The data, unless otherwise stated, were given as medians with IQRs for continuous variables and as simple frequencies (*n*) and percentages (%) for categorical variables. Student’s *t*-test and Mann–Whitney test were used for unpaired samples, as appropriate. Dichotomous variables were compared using Fisher’s exact tests or chi-squared test statistics, as appropriate. Binary logistic regression analysis was used to identify the demographic characteristics and risk factors for the pre-specified outcome examined. Survival was analysed by Kaplan–Meier curves and the statistical significance of the differences between the two groups was assessed using the log-rank test.

Multivariable logistic regression model was performed to tease out the independent predictors for BSI onset and for 28 day mortality in patients with BSI. The multivariate model was constructed using a forward stepwise procedure, entering all variables shown to be significant at the univariable analysis and those deemed clinically significant for the chosen outcome. Interaction effects between variables were also taken into consideration in the final model. *P* value analyses were two-sided and a *P* value of <0.05 was considered statistically significant. All statistical analyses were performed with Statistical Program for the Social Sciences (SPSS, version 22, SPSS Inc., Chicago, IL, USA) software.

### Ethics

The study was approved by the local Ethical Committee (no. 0341/2023), and informed consent was waived due to the observational nature of the research. The study was performed in line with the principles of the Declaration of Helsinki.

## Results

### General characteristics of study population

Overall, 129 patients were included in the study: 39 (30%) women and 90 (70%) men, with a median age of 64 (IQR 51–74) years. The general features of the study population are described in Table 1. Among the causes of ICU admission, the most common was respiratory failure, with 53 (41%) patients, followed by neurological disease (25; 19%) and polytrauma (20; 15.5%). All the enrolled COVID-19 patients presented with a critical illness with at least an acute respiratory distress syndrome condition and need for invasive or non-invasive ventilation. The median number of days from ICU admission to CRAB colonization was 12 (5–20) days, with the majority of patients (98%) receiving antibiotic therapy before colonization. Fifty-seven (44%) patients presented multisite CRAB colonization.

**Table 1.** General characteristics of study population and comparison between colonized-only and BSI patients

Characteristics	General population *N* = 129	Colonized-only patients *N* = 72	BSI patients *N* = 57	*P* value
**GENERAL, *N* (%)**				
*** *** Age, years, median (IQR)	64 (51–74)	65.5 (48.5–73.7)	61 (55–74)	0.344
*** *** Gender, female/male	39(30)/90(70)	24 (33)/48(77)	15(26)/42(74)	0.771
*** *** Department of hospitalization,				
*** *** Medical	35 (27)	18 (25)	17 (30)	0.556
*** *** Surgery	20 (15)	9 (12.5)	11 (19)	0.332
*** *** ICU	74 (58)	45 (62.5)	29 (51)	0.212
*** *** Cause of in-hospital admission,				
*** ***Lung diseases (including SARS-CoV-2 infection)	47 (36)	21 (29)	26 (45.5)	0.066
Heart diseases	6 (5)	1 (1.5)	5 (9)	0.209
*** ***Abdominal diseases	12 (9)	7 (9.5)	5 (9)	1.0
*** ***Kidney diseases	1 (1)	1 (1.5)	0 (0)	1.0
*** ***Neurological diseases	35 (27)	21 (29)	14 (24.5)	0.69
*** ***Haematological diseases	1 (1)	1 (1.5)	0 (0)	1
*** ***Sepsis or septic shock	2 (1.5)	2 (3)	0 (0)	0.502
*** ***Trauma/polytrauma	20 (15.5)	15 (21)	5 (9)	0.086
*** ***Other infection	1 (1)	0 (0)	1 (1.5)	0.442
*** ***Other	4 (3)	3 (4)	1 (1.5)	0.689
*** *** Cause of ICU admission				
*** ***Septic shock	5 (4)	4 (6)	1 (1.5)	0.382
*** ***Respiratory failure	53 (41)	24 (33)	29 (51)	**0**.**049**
*** ***Trauma/polytrauma	20 (15.5)	15 (21)	5 (9)	0.086
*** ***Cardiogenic shock/cardiac arrest	5 (4)	4 (6)	1 (1.5)	0.382
*** ***Neurological disease	25 (19)	16 (23)	9 (16)	0.381
*** ***Post surgery	18 (14)	8 (11)	10 (17.5)	0.205
*** ***Other	3 (2.5)	1 (1.5)	2 (3.5)	0.583
*** *** Type of ICU setting				
*** ***Multidisciplinary	34 (26)	23 (32)	11 (19)	0.113
*** ***COVID-19	50 (39)	21 (29)	29 (51)	**0**.**018**
*** ***Neurosurgery	29 (22.5)	18 (26)	11 (19)	0.526
*** ***Transplantation	2 (1.5)	1 (1.5)	0 (0)	1.0
*** ***Cardiothoracic surgery	1 (1)	1 (1.5)	1 (1.5)	1.0
*** ***Emergency room	13 (10)	8 (11)	5 (9)	0.773
**IN PREVIOUS 90 DAYS FROM ADMISSION, *N* (%)**				
*** *** Hospitalization	26 (20)	15 (21)	11 (19)	0.227
*** *** ICU admission	19 (15)	9 (13)	10 (18)	0.939
*** *** Antibiotic therapy	37 (29)	18 (25)	19 (33)	0.969
*** *** MDRO infection	4 (3)	1 (1.5)	3 (5)	0.713
*** *** Gram-negative MDR infection	3 (2.5)	2 (3)	1 (1.5)	0.797
*** *** Immunosuppression	7 (5.5)	4 (6)	3 (5)	0.960
*** *** Previous surgery	50 (39)	29 (40)	21 (37)	0.363
**COMORBIDITIES, *N* (%)**				
*** *** Myocardial infarction	12 (9)	3 (4)	9 (16)	0.356
*** *** Congestive heart failure	17 (13)	9 (13)	8 (14)	0.399
*** *** Hypertension	55 (43)	28 (39)	27 (47)	**0**.**034**
*** *** Obesity (BMI** ≥ **30** **kg/m^2^)	15 (12)	10 (14)	5 (9)	0.987
*** *** Cerebrovascular disease	16 (12)	10 (14)	6 (11)	0.242
*** *** COPD	12 (9)	6 (8)	6 (11)	0.851
*** *** Diabetes mellitus	22 (17)	11 (15)	11 (19)	0.185
*** *** Chronic kidney disease^a^	4 (3)	0 (0)	4 (7)	**0**.**025**
*** *** Solid tumour	19 (15)	8 (11)	11 (19)	0.952
*** *** Leukaemia/lymphoma	7 (5.5)	3(4)	4 (7)	0.238
*** *** CCI, median (IQR)	3 (1–5)	3 (1–4)	3 (1–6)	**0**.**041**
*** *** SAPS II score, median (IQR)	36 (27–49)	35 (26–49.5)	38 (29–46)	0.172
*** *** COVID-19 hospitalization	54 (42)	24 (33)	30 (53)	**0**.**014**
*** ***Need for CPAP/HFNC/NIV	45 (83)	20 (28)	25 (44)	0.367
Need for OTI	23 (43)	9 (13)	14 (25)	0.104
**CRAB COLONIZATION, *N* (%)**				
*** *** First colonization in ICU	123 (95)	70 (97)	53 (93)	0.396
*** *** Timing of colonization, median (IQR)				
*** ***From ER admission	18 (10–27)	17.5 (10–31.2)	18 (10–24)	**0**.**011**
*** ***From ICU admission	12 (5–20)	13 (4–21.2)	11 (5–18)	**0**.**016**
*** *** Site of colonization				
*** ***Respiratory tract	35 (27)	19 (26.5)	16 (28)	0.766
*** ***Rectal swab	93 (72)	52 (72)	41 (72)	1.0
*** ***Urine	1 (1)	1 (1.5)	0 (0)	0.987
*** *** Multisite	57 (44)	25 (35)	32 (56)	**0**.**003**
*** *** Number of sites				
*** ***1	72 (56)	47 (65)	25 (44)	0.082
*** ***2	49 (38)	21 (29)	28 (49)	0.059
*** ***3	8 (6)	4 (6)	4 (7)	0.679
*** *** Mechanical ventilation	81 (67)	39 (54)	42 (74)	**0**.**002**
*** *** ECMO	5 (4)	2 (3)	3 (5)	0.654
*** *** CRRT	9 (7)	3 (4)	6 (11)	0.240
*** *** Antibiotic therapy before colonization	127 (98)	71 (99)	56 (98)	0.321
*** *** Days of antibiotic therapy, median (IQR)	12 (4–21)	13 (5–21.7)	11 (3–20)	**0**.**044**
*** *** Steroids before colonization	74 (57)	42 (58)	32 (56)	0.427
*** *** Days of steroids, median (IQR)	11 (7–18)	11 (6.2–15.7)	12.5 (7–19)	0.242
*** *** CVC	115 (89)	63 (88)	52 (91)	0.897
*** *** Vesical catheter	127 (98)	70 (97)	57 (100)	0.446
*** *** Drainage	17 (13)	7 (10)	10 (17.5)	0.917
**OUTCOMES, MEDIAN (IQR)**				
*** *** Hospitalization length	45 (27–75)	44.5 (26–70)	51 (29.5–83)	0.097
*** *** ICU hospitalization length	30 (18–54)	24 (15.5–42)	33.5 (20–69)	**0**.**024**
*** *** IN-HOSPITAL ALL-CAUSE MORTALITY, *N*(%)	75 (58)	36 (50)	39 (68.5)	0.002
*** *** Mortality from colonization, *N* (%)				
*** ***7** **days	26 (20)	12 (17)	14 (25)	0.639
*** ***14** **days	33 (25)	15 (21)	18 (32)	0.098
*** ***28** **days	50 (39)	23 (32)	27 (47)	**0**.**036**
*** *** Days from colonization to mortality	19 (7–34)	17 (5.5–33)	21 (8.5–40.5)	0.587

CPAP, continuous positive airway pressure; HFNC, high-flow nasal cannula; NIV, non-invasive ventilation; OTI, orotracheal intubation; ECMO, extracorporeal membrane oxygenation; CRRT, continuous renal replacement therapy. Bold type indicates statistical significance.

a From moderate CKD (creatinine > 3 mg/dL) to dialysis or status post-kidney transplant.

**Figure 2.**
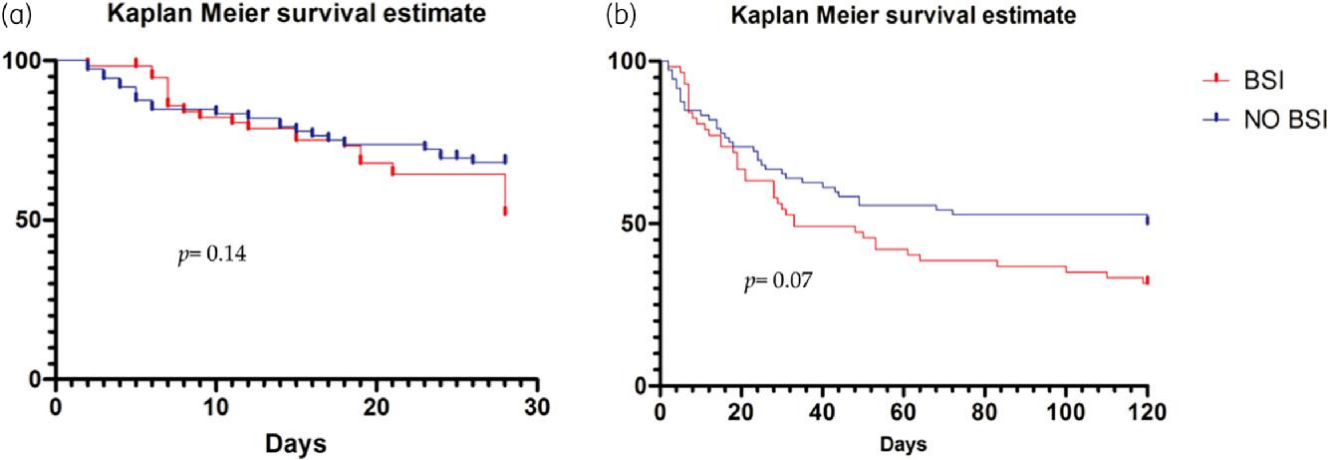
Cumulative proportions of all-cause 28 day (a) and overall (b) in-hospital mortality. The cumulative proportions of in-hospital mortality were estimated from CRAB colonization between colonized-only patients (no BSI) and BSI patients (BSI).

During hospitalization, 57 (44%) patients developed a CRAB BSI, whereas the remaining 72 (56%) did not. The overall in-hospital mortality was 58%, being 50% and 68.5% in the colonized-only and in BSI patients, respectively (*P* = 0.002). As for the 28 day mortality rate from CRAB colonization, we observed a statistically significant difference between colonized-only patients and BSI patients (32% versus 47%, respectively, *P* = 0.0036). However, no significant difference was observed for the cumulative survival analyses on 28 day and on overall in-hospital mortality. Of note in the overall mortality, there was a trend toward higher mortality in BSI patients than in colonized-only ones (Figure 2).

### Risk factors for BSI onset

Univariate analysis between colonized-only patients and BSI patients showed significant differences in terms of CCI (*P* = 0.041), hypertension (*P* = 0.034), COVID-19 (*P* = 0.014), colonization time from ICU admission (*P* = 0.016), need for mechanical ventilation at the time of colonization (*P* = 0.002), multisite colonization (*P* = 0.003) and days of antibiotic therapy prior to colonization (*P* = 0.044), whereas no significant difference was observed when considering the subgroups of single antibiotic classes used prior to colonization (data not shown). In multivariate analyses, an increased CCI (*P* = 0.026), COVID-19 (*P* < 0.001), mechanical ventilation (*P* = 0.012) and multisite colonization (*P* = 0.012) were independently associated with BSI onset (Table 2a).

**Table 2.** Multivariable analysis

Risk factors	OR (95% CI)	*P* value
**Risk factors for BSI onset in patients with CRAB colonization**		
*** *** CCI	1.34 (1.02–15.2)	**0**.**026**
*** *** COVID-19	2.32 (1.72–15.8)	**<0**.**001**
*** *** Hypertension	1.87 (0.91–3.87)	0.089
*** *** SAPS II	2.5 (0.88–11.5)	0.091
*** *** Timing of ICU to colonization	1.2 (0.84–9.9)	0.122
*** *** Multisite >1	2.4 (1.2–4.90)	**0**.**016**
*** *** Mechanical ventilation	2.34 (1.1–5.02)	**0**.**024**
**Mortality risk factors in the BSI patients**		
*** *** Age	1.9 (1.72–15.2)	**0**.**026**
*** *** CCI	2.41 (1.7–15.8)	**<0**.**001**
*** *** COVID-19	1.2 (0.82–8.8)	0.122
*** *** SAPS II	2.5 (0.87–11.5)	0.091
*** *** PMN/LYM	1.2 (0.81–9.9)	0.122
*** *** Steroids	0.9 (0.8–7.98)	0.765
*** *** BSI CVC-related	0.7 (0.4–11.87)	0.455
*** *** Source control	0.6 (0.1–16.6)	0.217
*** *** Early active therapy	0.32 (0.02–0.68)	**0**.**002**
*** *** Septic shock	1.5 (1.41–9.7)	**0**.**001**
*** *** Pitt score	2.7 (1.6–14.4)	**<0**.**001**
*** *** FUBCs	1.14 (0.92–20.1)	0.077
*** *** Clinical improvement at 72** **h	0.4 (0.2–0.79)	**0**.**011**

Multisite >1, patients with more than one colonization; PMN/LYM, neutrophil/lymphocyte ratio; early active therapy, use of at least one *in vitro* active drug within the first 24 h from the BSI onset; FUBCs, follow-up blood cultures performed. Bold type indicates statistical significance.

### Patients with CRAB BSI

**Figure 3.**
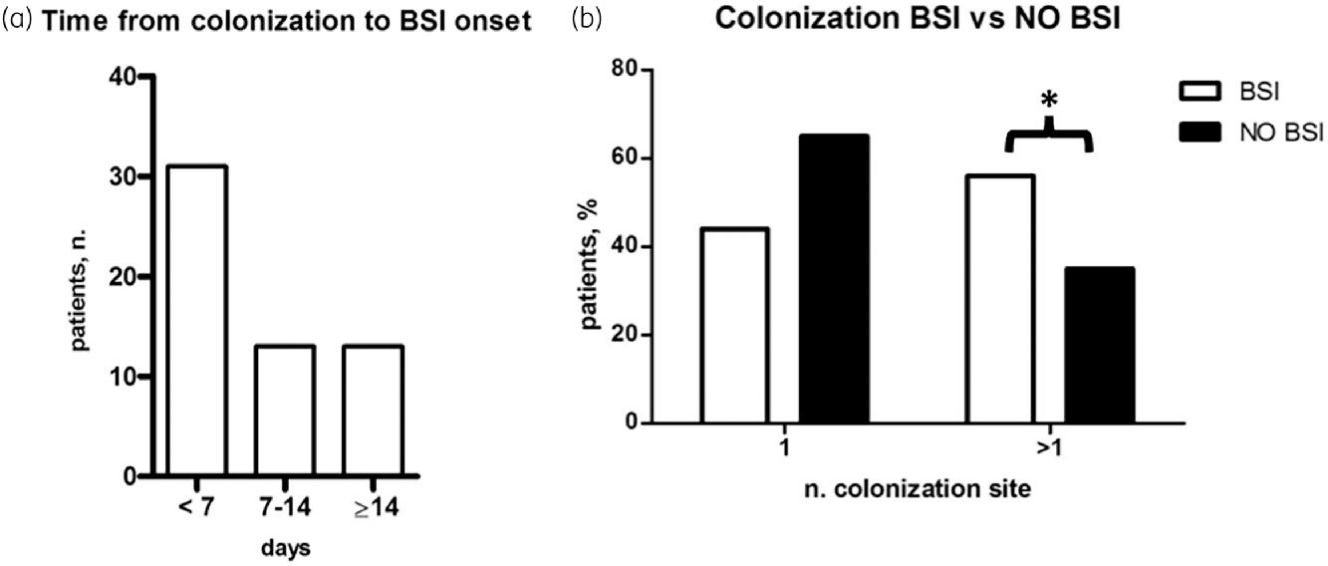
CRAB colonization in BSI patients. (a) Timing from colonization to BSI onset in the BSI patients group. (b) Relationship between single versus multisite colonization in the no BSI patients and BSI patients. * *P* < 0.05.

The characteristics of the BSI group are described in Table 1 and Table 3. The median time from colonization to BSI onset was 6 (0–10) days (Figure 3a); the majority of BSI patients (31; 54%) showed BSI onset within the first week from colonization. The patients were more likely to present rectal colonization (41; 72%), while 32 patients (56%) showed multisite colonization. The features of the number of colonization sites and the comparison between BSI and colonized-only patients are described in Figure 3b.

**Table 3.** General characteristics of BSI population and comparison between survivors and non-survivors at 28 days from BSI onset

Characteristics	BSI patients *N* = 57	28 day survivors *N* = 30	28 day non-survivors *N* = 27	*P* value
**GENERAL, MEDIAN (IQR)**				
*** *** Age, years	61 (55–74)	59 (50.25–71.2)	66 (59–76)	**0**.**038**
*** *** Gender, female/male, *N* (%)	15(26)/42(74)	10 (33)/20 (67)	5 (19)/22(81)	0.207
*** *** Timing of colonization				
*** ***From ER admission	24 (16–37)	27 (16–41.5)	21 (16.5–28)	0.186
*** ***From ICU admission	19 (10–29)	24.5 (12–39)	15 (8.5–2-.5)	0.053
**COMORBIDITIES, *N* (%)**				
*** *** CCI, median (IQR)	3 (1–6)	2 (1–3)	4 (2–7)	**0**.**024**
*** *** SAPS II score, median (IQR)	38 (29–46)	33.5 (26.2–41)	44 (36.5–50.5)	**0**.**050**
*** *** COVID-19 hospitalization	29 (53)	10 (33)	19 (70)	**0**.**010**
**Clinical features at BSI onset, *n* (%)**				
*** *** Inotropic support	23 (40)	7 (23)	16 (59)	0.006
*** *** Mechanical ventilation	40 (70)	18 (60)	22 (8)	0.076
*** *** ECMO	3 (5)	0 (0)	3 (11)	0.083
*** *** CRRT	7 (12)	2 (7)	5 (19)	0.191
*** *** Pitt score, median (IQR)	5 (2–8)	3 (1.25–6)	8(4–8)	**0**.**001**
*** *** Septic shock	17 (30)	3 (10)	14 (52)	**0**.**001**
*** *** Source of infection				
*** ***Lung (no VAP)	6 (10.5)	1 (3)	5 (19)	0.076
*** ***VAP	18 (31.5)	9 (30)	10 (37)	0.583
*** ***Urine tract	1 (1.5)	1 (3)	0 (0)	0.326
*** ***Skin and soft tissue/wound	1 (1.5)	1 (3)	0 (0)	0.326
*** ***Primary BSI	20 (35)	9 (30)	11 (41	0.407
*** ***CVC-related	11 (19)	9 (30)	1 (4)	**0**.**007**
*** *** Source control	12 (21)	10 (33)	2 (7)	**0**.**012**
**Laboratory findings at BSI onset, median (IQR)**				
*** *** PMN **×** 10^9^	7.72 (5.2–14)	7.1 (5.2–10.2)	9.14 (6.4–15.6)	0.103
*** *** LYM × 10^9^	0.76 (0.5–1.07)	0.94 (0.68–1.16)	0.6 (0.4–0.86)	**0**.**026**
*** *** PMN/LYM	10.7 (5.3–25.6)	9.1 (3.92–13.6)	13.6 (8.3–30.3)	**0**.**030**
*** *** CRP, mg/dL	14.1 (7.6–22.2)	13.5 (4.65–20.5)	14.5 (10.4–24.2)	0.564
*** *** PCT, ng/mL	0.58 (0.19–3.4)	0.37 (0.197–3.9)	0.87 (0.19–3.43)	0.817
*** *** Albumin, g/dL	2.6 (2.5–3)	2.6 (2.5–3)	2.7 (2.4- 3.1)	0.808
**Microbiological data, *n* (%)**				
*** *** MDR resistance profile	9 (16)	4 (13)	5 (18)	0.722
*** *** XDR resistance profile	48 (84)	26 (87)	22 (82)	0.722
*** *** Colistin resistant	9 (16)	2 (7.5)	7 (26)	0.070
*** *** Availability of cefiderocol susceptibility	46 (81)	22 (73)	24 (88)	0.186
*** *** Of which cefiderocol resistant	2 (4.5)	1 (7)	1 (3)	1.0
**Treatment data, *n* (%)**				
*** *** Early active therapy	38 (65.5)	23 (77)	15 (56)	**0**.**034**
*** *** Time to definitive therapy, median (IQR)	1 (1–2)	1 (1–1.5)	1 (0.25- 3)	0.838
*** *** Definitive therapy within 48 h	31 (54)	19 (63)	12 (44)	0.189
*** *** Definitive appropriate therapy	45 (79)	25 (83)	20 (74)	0.519
*** *** Monotherapy	11 (19)	6 (20)	5 (19)	0.828
*** *** Combination therapy	40 (70)	21 (70)	19 (70)	0.934
*** *** Regimen based on				
*** ***Colistin	41 (72)	23 (77)	18 (67)	0.413
*** ***Cefiderocol	14 (24.5)	7 (23)	7 (26)	0.825
*** ***Fosfomycin	29 (51)	13 (43)	15 (59)	0.237
*** ***Ampicillin/sulbactam	18 (31.5)	9 (30)	8 (30)	0.976
**Outcomes, *n* (%)**				
*** *** Microbiological outcomes, FUBC performed	39 (68.5)	24 (80)	15 (56)	**0**.**042**
*** *** FUBC negative at:				
*** ***72 h	7 (12.5)	4 (13)	3 (11)	0.788
*** ***7 days	26 (45.5)	16 (53)	10 (37)	0.952
*** ***14 days	36 (63)	22 (73)	14 (52)	0.798
*** *** Clinical improvement at 72 h	17 (30)	15 (50)	2 (7)	**0**.**001**
*** *** Clinical cure	28 (49)	26 (87)	2 (7)	**0**.**001**
BSI recurrence at 30 days	5 (9)	3 (10)	2 (7)	0.705
*** *** New CRAB infection, no BSI (after BSI)	14 (24.5)	12 (40)	2 (7)	**0**.**003**
*** *** Other MDRO infection at 30 days	17 (30)	15 (50)	2 (7)	**0**.**002**
*** *** ICU hospitalization length, median (IQR)	33.5 (20–69)	64 (34–81)	22 (18–32)	**<0**.**001**
*** *** Hospitalization length, median (IQR)	51 (29.5–83)	73.5 (54.7–122.2)	31 (20.5–43)	**<0**.**001**
*** *** Mortality from BSI onset				
*** ***7 days	14 (25)	0 (0)	14 (52)	**0**.**001**
*** ***14 days	18 (32)	0 (0)	18 (67)	**0**.**001**
*** ***28 days	27 (47)	0 (0)	27 (100)	**<0**.**001**
*** ***In-hospital all cause mortality	39 (68.5)	12 (43)	27 (100)	**0**.**001**

ER, Emergency Department; ECMO, extracorporeal membrane oxygenation; CRRT, continuous renal replacement therapy; PMN, neutrophils; LYM, lymphocytes; PMN/LYM, neutrophil/lymphocyte ratio; CRP, C-reactive protein; PCT, procalcitonin; FUBC, follow-up blood culture. Bold type indicates statistical significance.

Primary bacteraemia was the BSI source in 20 (35%) cases, followed by 18 (31.5%) cases with ventilator-associated pneumonia (VAP) and 11 (19%) cases of central venous catheter (CVC)-related BSI. As for microbiological data, 84% of the *A. baumannii* strains isolated showed an XDR resistance profile; resistance to colistin was demonstrated in 9 (16%) cases, while 2 (4.5%) out of 46 (81%) strains tested for cefiderocol showed *in vitro* resistance. As for treatment regimens, colistin was the only drug used in monotherapy (11 patients, 19%) and the most commonly used drug in combination regimes (30 patients, 75%), with the combination colistin + fosfomycin used in almost 25% of patients. Appropriate therapy in the first 24 h after the development of BSI was administered in 65.5% of patients, while definitive therapy was effective in 79%, with almost 55% of patients receiving it in the first 48 h.

### Risk factors for 28 day mortality in patients with BSI

In the BSI population, we assessed risk factors for 28 day mortality. Characteristics of the surviving and non-surviving BSI patients are described in Table 3. On multivariate analysis, we observed that age (*P* = 0.021), an increased CCI (*P* < 0.001), septic shock (*P* = 0.001) and Pitt score (*P* < 0.001) were factors independently associated with mortality, while early appropriate therapy within 24 h (*P* = 0.002) and clinical improvement at 72 h (*P* = 0.011) demonstrated a protective prognostic effect on outcome (Table 2b).

## Discussion

In this study, we observed that 44% of CRAB-colonized patients admitted to an ICU subsequently developed a CRAB BSI during hospitalization, more frequently in the first 7 days from colonization. Burden of comorbidities (expressed by the CCI), COVID-19, multisite colonization and the need for mechanical ventilation were risk factors for CRAB BSI. In addition, we confirmed that the patients’ comorbidities, as well as clinical severity at bacteraemia onset, were the most significant prognostic factors of poor outcome in BSI patients, whereas the prompt start of appropriate antibiotic therapy was a predictor of survival.

To the best of our knowledge, this is the first study in the COVID-19 era that explored risk factors for developing BSI in critically ill patients with previous CRAB colonization.

The role of CCI and pneumonia, particularly VAP, as factors associated with BSI onset has been already demonstrated.^16,18,21^ Although mechanical ventilation is a known risk factor for CRAB colonization and infection, often associated with pneumonia, in our study population we have observed how ventilation *per se* is associated with BSI development.^31^ A rather interesting aspect of our research is that we have identified the importance of COVID-19 and multisite colonization as risk factors for CRAB BSI. Indeed, our study confirms the negative impact of COVID-19 on patients admitted to the ICU, predisposing them to the development of invasive infections with high mortality rates.^8,9^ The relationship between SARS-CoV-2 infection and CRAB BSI onset could be explained by translocation phenomena both at the respiratory tract level, due to the severe pneumonia, and at the intestinal level, related to the combined effect of hypoxic and direct virus damage. Indeed, several authors have pointed out a relevant dysfunctional gut mucosal barrier in COVID-19 severe patients due to the expression of ACE-2 on enterocytes, which might act as an entrance site for SARS-CoV-2, and high IL-6 levels, which may promote and maintain systemic inflammation causing, at the end, the so-called cytokine storm typical of severe COVID-19 infection.^32^ Furthermore, recent reports showed that low-grade endotoxaemia is detectable in patients with COVID-19 and is associated with thrombotic events, probably due to activation of NOX-2, increased oxidative stress, low bioavailability of nitric oxide and endothelial dysfunction.^33,34^ A change in the microbiota and persistent microbial translocation has been observed during severe SARS-CoV-2 infections, a condition that could play a pathogenetic role in BSI development during COVID-19, a hypothesis supported by the high rate of primary bacteraemia that we observed.^33,35,36^ A recent study evaluated the possible role of a persistent high level of microbial translocation as the pathogenetic trigger for the development of primary BSI following *Clostridioides difficile* infection (CDI); the authors found that patients who developed primary BSI maintained high levels of LPS-binding protein (LBP) and low levels of EndoCAb IgM, both markers of microbial translocation, while patients who did not develop BSI showed a reduction of microbial translocation levels, similar to that of healthy donors.^37^

On the other hand, the impact of the pandemic on the healthcare systems, with its interference in infection control and antibiotic stewardship protocols, could have affected the diffusion of CRAB colonization and infection in ICU settings, as recently shown.^10,11,38^

However, one of the most important findings of the present research was the role of multisite colonization as an independent predictor of BSI. Indeed, while its importance had been previously demonstrated in *Enterobacterales* BSI, being incorporated in the Giannella score,^14^ no data regarding CRAB BSI exist so far. It could be speculated that the burden of colonization, expressed as the presence of CRAB at different sites, and the homeostasis alteration in particular organs, such as the gut and the lungs, may favour bacterial translocation and subsequent BSI onset.

Although the use of antibiotic therapy, particularly β-lactams, was a known risk factor for infection in previous studies, in our research, neither duration nor specific classes of antibiotics reached statistical significance as risk factors for BSI.^19,20,39^

Thus, following the results of our study, a patient colonized by CRAB is at highest risk of BSI development if colonization is localized at multiple sites and in the presence of a high burden of comorbidities at baseline, SARS-CoV-2 infection and mechanical ventilation. Furthermore, the first week after CRAB colonization seems to be the most vulnerable period. These factors could play a broader role in terms of infection control and antimicrobial stewardship programmes. As a matter of fact, on the one hand, highlighting which patients are at higher risk of BSI onset could help clinicians to start prompt appropriate therapy with an improvement in the clinical outcome; on the other hand, by understanding which patients are at low risk of BSI development, the use of broad-spectrum antibiotics active against CRAB could be avoided, with a reduction in consumption of WHO ‘reserve’ class drugs.

We confirmed that mortality in colonized-only or BSI patients remains very high.^3,19,39,40^ In our BSI patients, we found a 28 day mortality rate of 47%, similar to that published recently (42%) but lower than that observed previously in another study (73%).^4,7^

In BSI patients, we confirmed that the factors associated with mortality are age, the burden of comorbidities stratified by CCI, and clinical severity at BSI onset, defined in terms of Pitt score and septic shock. These risk factors have been demonstrated in numerous studies on *A. baumannii* and validated as prognostic factors for *Enterobacterales* BSIs in the INCREMENT score.^4,15,19–21,40^ However, no significant association was observed between mortality and clinical severity on ICU admission (SAPS II score), source of BSI, control of the source of infection, use of steroids, or prolonged hospitalization, as shown in other studies.^4,16,41,42^ A recent study reported risk factors for infection-related mortality in *A. baumannii*, dividing them into modifiable and non-modifiable factors. Improving the management of the modifiable risk factors (such as hospital stay, duration of antibiotic therapy, invasive procedures or ICU admission) should be key to improving clinical outcomes in these difficult-to-treat infections, as has been already described.^42^

COVID-19 did not have a direct negative impact on mortality either, despite the statistical significance observed in the univariate analysis between patients who survived and died by 28 days. Thus, COVID-19 does not appear to have had a direct impact on mortality in our study population, representing only a risk factor for the development of BSI. This is a noteworthy finding, which provides additional data about the relationship between COVID-19 and CRAB infection, a great challenge for clinicians in these pandemic years.^10^

Early appropriate therapy and related clinical improvement within 72 h are confirmed as important protective factors; however, in our study no specific drug regimen has been associated with survival.^22,39,43^ Indeed, neither colistin- nor cefiderocol-based regimens demonstrated higher survival rates and not even combination therapy compared with monotherapy achieved a significant difference between the two groups. Although these results are in line with the lack of strong scientific evidence in terms of optimal therapy for CRAB infections reported by the recent European guidelines, our study population is certainly too small to be able to analyse the efficacy of different treatment regimens, and further studies are needed to investigate this issue.^44^

Our study undoubtedly presents important limitations that should be acknowledged. Firstly, the observational nature of the study and the relatively small sample size of patients with BSI did not allow an exhaustive analysis in terms of BSI onset and risk factors for mortality as well as the effectiveness of the drug regimens used. Secondly, the retrospective data extraction afterwards to complete the data entry could have influenced the prospective nature of the study design, leading to some biases. Thirdly, having enrolled patients in the COVID-19 era makes it more complex to compare our findings with previous studies and to generalize our results to settings without SARS-CoV-2 infections. However, important relationships emerged between COVID-19 and CRAB, which will certainly have future clinical implications and require further studies.

We decided to exclude patients with expected survival less than 48 h from colonization in order to avoid bias unrelated to the CRAB colonization, but rather, related to the highly compromised clinical conditions of the patients. Indeed, by including colonized patients who died within 48 h, the associated risk factors for BSI development would have been probably biased by the patients’ conditions.

Finally, the single-centre nature of the study, reflecting the ecology and clinical management of intensivists and Infectious Disease consultants in our hospital, requires confirmation in further centres with similar colonization and CRAB infection epidemiology. Definitely, a larger and multicentre study will be essential to provide robust and generalizable results.

In conclusion, we showed that multisite colonization, burden of comorbidities, COVID-19 and need for mechanical ventilation are risk factors for CRAB BSI onset in critically ill patients with CRAB colonization. Of note, BSI occurred more frequently in the first 7 days from colonization.

These predictors for CRAB BSI could be useful to identify patients at highest risk of BSI, prompting clinicians to start the most appropriate empirical therapy early. Should our results be confirmed in future studies, these factors could become useful tools in antibiotic stewardship protocols.

## Data Availability

The datasets used and/or analysed during the current study are available from the corresponding author on reasonable request.
